# The role of multislice computed tomography (MSCT) angiography in the diagnosis and therapy of non-occlusive mesenteric ischemia (NOMI): Could MSCT replace DSA in diagnosis?

**DOI:** 10.1371/journal.pone.0193698

**Published:** 2018-03-01

**Authors:** Sara Kammerer, Christoph Schuelke, Shoma Berkemeyer, Aglae Velasco, Walter Heindel, Michael Koehler, Boris Buerke

**Affiliations:** 1 Department of Clinical Radiology, University Hospital Muenster, Muenster, Germany; 2 Reference Center for Mammography, University Hospital Muenster, Muenster Germany; Medical University Innsbruck, AUSTRIA

## Abstract

**Objectives:**

Evaluation of multislice-CT (MSCT) during diagnosis and therapeutic decision-making in patients with suspected non-occlusive mesenteric ischemia (NOMI).

**Methods:**

Retrospective, institutional review board-approved study of 30 patients (20 men, 10 women, mean age 64.6±14.2 years, range 24–87 years) undergoing biphasic abdominal MSCT followed by digital subtraction angiography (DSA) due to suspected NOMI. MSCT and DSA were qualitatively and quantitatively evaluated independently by two radiologists with respect to the possible diagnosis of NOMI. MSCT analysis included quantitative measurements, qualitative evaluation of contrast enhancement and assessment of secondary findings (bowel wall thickening, hypo-enhancement, intestinal pneumatosis). MSCT diagnosis and secondary findings were compared against DSA diagnosis.

**Results:**

NOMI was diagnosed in a total of n = 28 patients. No differences were found when comparing the R1-rated MSCT diagnosis (p = 0.09) to the “gold standard”, while MSCT diagnosis was slightly inferior with R2 (p = 0.02). With R1, vessel-associated parameters revealed the best correlation, i.e. qualitative vessel width (r = -0.39;p = 0.03) and vessel contrast (r = 0.45;p = 0.01). Moderate correlations were found for quantitative vessel diameters in the middle segments (r = -0.48,p = 0.01), increasing to almost high correlations in the distal (r = -0.66;p<0.00001) superior mesenteric artery (SMA) segments. No significant correlation was apparent from secondary findings.

**Conclusions:**

MSCT is an appropriate non-invasive method for diagnosing NOMI and leads to adequate and immediate therapeutic stratification.

## Introduction

Acute mesenteric ischemia is a life-threatening perfusion disorder of the intestine that can be caused by arterial (85%-95%) or venous (5%-15%) pathologies. In terms of arterial pathologies, occlusion from embolism accounts for 40%-50%, from thrombosis 20%-30%, and the non-occlusive form for approximately 25% [1–3] of all events.

The pathophysiological correlates for non-occlusive mesenteric ischemia (NOMI) are severe vasospasms of the mesenteric arteries, often triggered by generalized shock with reduced cardiac output and activated sympathetic nervous system, often aggravated by the need for vasoconstrictive medication. Until irreversible wall damage occurs, the symptoms can alternate in intensity (i.e., colic-like pain) and be unspecific. Most often, (sub)ileus may be diagnosed [4, 5].

While occlusion of the mesenteric artery from embolism or thrombosis has been studied closely, with numerous recent publications [6–9], NOMI does not receive much attention despite its poor prognosis, reaching mortality rates of approx. 50% [10–12]. Probable explanations for such high mortality can be advanced age along with severe comorbidities [13] and delayed diagnosis. The main reasons for delayed diagnosis are considered to be the unspecific clinical symptoms and diagnostic findings on clinical examination, along with the often reluctant decision to conduct an invasive diagnostic procedure, namely angiography [4, 14, 15].

At present, digital subtraction angiography (DSA) is accepted as the “gold standard” in the diagnosis and treatment of NOMI [11]. Aside from its invasive nature, DSA might not always be available everywhere. Therefore, the increasing availability of rapidly improving MSCT scanners suggests that the current diagnostic algorithms should be re-evaluated [16]. Furthermore, multislice CT (MSCT) permits visualization of potentially severe abdominal findings. MSCT with non-invasive MSCT angiography could be a faster means of distinguishing patients in need of immediate surgery from those in whom therapeutic DSA may be sufficient but urgently required [17, 18]. Considering the intestine’s ischemic tolerance of only 3–6 hours before irreversible damage occurs, the primary goal should be a fast but reliable diagnostic workup.

The purpose of our study, therefore, was to compare MSCT and DSA in the diagnosis of suspected NOMI with a view to therapeutic decision-making.

## Material and methods

The study has been approved by the local IRB (Westfalen-Lippe, Muenster, Germany). Informed consent has been obtained from all patients.

### Patients

A total of 30 patients (20 men, 10 women) with a mean age of 64.6 ± 14.2 years (range 24–87 years) were included in this institutional review board-approved, retrospective analysis covering the period September 2009 to January 2015. All patients, where NOMI was clinically suspected due to (colic-like) abdominal pain, partly followed by a painless interval, in some cases non-specific symptoms such as nausea and vomiting and rapidly increasing lactate with an anamnesis of recent surgery or high adrenaline levels, underwent biphasic (arterial und venous phase) abdominal MSCT followed by DSA within 24 hours (median 1:37 hours, maximum 13:56 hours due to the need for circulatory stabilization and prior operation). The morbid conditions were caused by cardiac surgery for 18 patients, liver transplantation for 2 patients, kidney transplantation for 1 patient and a heart transplantation for 1 patient. Further, one patient suffered from acute renal failure, 4 patients received intestinal surgery and 5 patients needed ICU-treatment (of these, 2 caused by trauma and 3 by a septic course of disease). Written informed consent was obtained from every patient prior to the examination, if possible.

During that period, 12 patients received arterial phased CT of the abdomen with reported suspicion of NOMI without being examined with DSA subsequently. In two cases, additional diagnostic or therapeutical procedures were refused by the patients, in 4 cases the diagnostic or clinical condition were severe to an extent, that no further steps were taken and 6 patients underwent immediate laparotomy in which the diagnosis could be verified. One patient showed a rapid decline of his increased lactate levels, the other 5 patients with suspected NOMI did not undergo laparotomy due to refusal or premature death, mostly caused by sepsis or multiorganfailure.

### Data acquisition

The examinations were performed using a dual source 128-slice CT scanner (Somatom^®^ Definition Flash, Siemens AG, Medical Solutions, Forchheim, Germany). The i.v. contrast agent (Ultravist^®^-370, Bayer Schering Pharma, Leverkusen, Germany) was applied at a constant injection rate of 3 mL/s. The arterial contrast phase was determined by bolus tracking. The venous contrast phase was defined at 75 seconds after i.v. contrast agent injection. Images were obtained at a tube voltage of 120 kV with a collimation of 32 x 0.6 mm, using a special dose-modulation template (CARE dose4DTM, Siemens AG, Medical Solutions, Forchheim, Germany) to reduce radiation exposure [19, 20]. In some cases water was administered as a negative oral contrast before CT acquisition [21].

### Digital subtraction angiography

Digital subtraction angiography was performed using a monoplanar angiography suite (Philips Healthcare, Best, Netherlands). The superior mesenteric artery (SMA) was selectively probed via transfemoral artery access. Complete angiography of the SMA supply zone was performed by administering standardized contrast agent (Ultravist 300) at a volume of 15 mL and a flow rate of 5 mL per second. On confirming the possible NOMI diagnosis, selective intra-arterial (i.a.) prostavasin therapy was initiated in accordance with Ernst et al. [4, 17, 22] using an initial bolus of 10 mg followed by a maintenance dose of 2 mg per hour.

### Data preparation

The data were prepared by someone not involved in the later evaluation process. All CT data sets were reconstructed at a slice thickness of 1.5 mm with a reconstruction increment of 0.6 mm in transverse and coronal orientation [18]. Angiographic data were stored as series with and without digital subtraction. All examinations were anonymized and transferred to a picture archiving and communication system (PACS) (CentricityTM PACS, GE Healthcare, Fairfield, Connecticut, USA); the CT data were additionally sent to a rendering server for further multiplanar reconstructions and maximum intensity projections using Syngovia^®^ (Siemens Healthcare, Forchheim, Germany).

### Data evaluation

Two radiologists (R1 with eight years of professional experience including performing DSA examinations, as well as extensive experience in evaluating cardiovascular CT- and MRI-examinations and R2 with four years of experience in abdominal MSCT imaging) independently reviewed and analyzed the anonymized MSCT and DSA images in random order with no time limit or other constraints. Besides evaluation of the predefined orientations, the vessel diameters were quantified in curved multiplanar reconstructions using the above-mentioned software tools [23]. The following key points were taken as the basis for evaluation:

#### Diagnosis of NOMI

Both readers rated the MSCT examinations for the presence of NOMI on a four-point scale (definitely, probably, probably not, definitely not).

#### Visual assessment of mesenteric vessel width and vessel contrast

Arterial and venous mesenteric vessel width was scored using a four-point scale (barely definable, narrow, regular or dilated vessel diameter). Vessel contrast enhancement was qualitatively assessed on a three-point scale (strongly, moderately or barely visible contrast).

#### Quantitative measurement of vessel diameters

The vessel diameters orthogonal to the vessel’s centerline were quantified in a standardized manner, by taking measurements at predefined positions:

- proximal superior mesenteric artery (SMA), 1 cm distal to the origin
- middle SMA after the origin of the first segmental branch
- distal SMA territory, represented by the proximal ileocolic artery
- superior mesenteric vein (SMV), 1 cm proximal to the portal vein

#### Evaluation of secondary MSCT signs

The presence or absence of potential secondary signs of ischemic bowel damage were rated and correlated to the definite diagnosis. Specifically, the secondary signs comprised bowel wall edema, bowel wall thickening, bowel wall hypoenhancement, bowel loop distension, intestinal pneumatosis, portal gas accumulation, mesenteric edema, vessel irregularities and atherosclerosis.

#### Definition of “gold standard”

Applying the “gold standard”, the angiographic data were analyzed by a specialized interventional radiologist with 15 years’ experience in angiography. A positive diagnosis was verified, moreover, by the clinical course (vessel-diameter normalization, reduction of symptoms and decrease in lactate under i.a. prostavasin therapy, intraluminal findings in case of endoscopy, intraoperative findings in case of laparotomy).

### Statistical analyses

Excel 10.0 (Microsoft Corporation, Redmond, USA) and SAS 9.3 (SAS Institute Inc., Cary, USA) were used for statistical analysis. P ≤ 0.05 was considered significant. Difference in readers was tested using Student’s t-test, Fisher’s test and Mann-Whitney test. Pearson’s and Spearman’s correlation were used to analyze the association between the readers.

## Results

### Diagnosis of NOMI

NOMI was diagnosed in 28 MSCT and 28 DSA cases. Two cases of NOMI detected by DSA were not found on prior MSCT, and 2 cases of NOMI were suspected from MSCT but ruled out by DSA. In 12 patients (38,7%) radiologic diagnosis was confirmed by explorative surgery. A total of 21 patients (67,74%) died during the course of the disease and were autopsied, so that the diagnosis could also be confirmed.

The Mann-Whitney test revealed no difference in performance when comparing the R1 results for MSCT diagnosis (p = 0.09) against the “gold standard”, and an inferior performance with low correlation in the case of R2 (p = 0.02).

### Visual assessment of mesenteric vessel width and vessel contrast

In the case of R1, vessel-associated parameters revealed a low (negative) correlation between qualitative arterial and venous vessel width (r = -0.39, p = 0.03) and a moderate correlation for vessel contrast (r = 0.45, p = 0.01). The negative relationship in qualitative vessel width indicates that a smaller vessel diameter is correlated with the likely diagnosis of NOMI. The same applies to reduced vessel contrast. With R2, no correlations were found between qualitative vessel width (r = 0.09, p = 0.64) and vessel contrast (r = 0.11, p = 0.56).

### Quantitative measurement of vessel diameters

With respect to the SMA diameters measured by R1, a moderate correlation to the “gold standard” diagnosis of NOMI was found for the middle segment (r = -0.48, p = 0.01) with an increasing correlation for the distal segment (r = -0.66, p<0.00001). In addition, a moderate correlation was found for the SMV diameter (r = 0.4, p = 0.02).

The mean SMA diameters for both readers were 5.9 mm (± 1.4 mm) in the proximal, 4.3 mm (± 1.5 mm) in the middle and 2.6 mm (± 1.1 mm) in the distal segment, respectively. The mean diameter of the SMV was 9.2 mm (± 2.9 mm); see Table 1.

**Table 1:** Measurements of arterial and venous mesenteric vessel diameters.

	Reader 1 and reader 2	Reader 1	Reader 2
**P-SMA**	5.85 (1.38)	5.87 (1.53)	5.83 (1.38)
**M-SMA**	4.52 (1.54)	4.23 (1.78)	4.80 (1.48)
**D-SMA**	2.56 (1.14)	2.88 (1.49)	2.23 (1.27)
**SMV**	9.20 (2.94)	8.30 (4.20)	10.09 (2.65)

Mean ± standard deviation (SD), (mm), of the proximal superior mesenteric artery (P-SMA) 1 cm distal to the origin, the middle SMA (M-SMA) after the origin of the first segmental branch, the distal SMA territory (D-SMA) represented by the proximal ileocolic artery, and the superior mesenteric vein (SMV) 1 cm proximal to the portal vein.

### Evaluation of secondary MSCT signs

The incidence of secondary signs, as recorded by both readers, was 26.7% for bowel wall edema, 36.7% for bowel wall thickening, 46.7% for bowel wall hypo-enhancement, 23.3% for bowel loop distension, 6.7% for intestinal pneumatosis, 0% for portal gas accumulation, 76.7% for mesenteric edema, 46.7% for vessel irregularities, and 60.0% for atherosclerosis.

A statistically significant difference between the readers was only found in the case of abdominal wall thickening (p = 0.004) and blood vessel irregularities (p = 0.02).

Except for atherosclerosis (r = 0.32, p = 0.08), none of these signs were close to statistically significant.

## Discussion

NOMI is a severe complication in patients with generalized shock, which results in reduced cardiac output and an activated sympathetic nervous system. Due to unspecific symptoms, largely consistent with (sub)ileus and unspecific laboratory values, NOMI is often diagnosed late [24–26]. Aside from the severity of the disease itself, delayed diagnosis is responsible for the poor prognosis, with a mortality rate of more than 50% [14].

Early diagnosis with the aid of a non-invasive imaging method such as MSCT for therapeutic stratification would thus be appropriate, especially if DSA is not always available.

However, there are no standardized MSCT criteria for diagnosis of NOMI at present and the significance of different imaging features in MSCT has not ultimately been validated.

In our series, diagnosis of NOMI with MSCT was equivalent to DSA when the grade of vessel enhancement (arterial and venous) around the bowel and the diameter of the AMS were assessed by experienced radiologists. According to the results of Bourcier et al.[26], secondary findings such as advanced ischemia were not significant in our study, because most of these cases underwent surgery immediately and therefore were excluded.

As revealed by this study, the diagnosis of NOMI can be confirmed by an experienced reader in MSCT as compared to DSA as the “gold standard.” Experience in this specific diagnosis is essential in ensuring reliable image analysis, as evidenced by the slightly inferior performance of the less experienced reader.

The most significant imaging criteria upon which the diagnosis is based are vessel contrast and vessel diameter, where determination of the diameter can be either qualitative or quantitative. Anyhow, no anatomical threshold for differentiating between the normal and pathological vessel width of the mesenteric arteries can be found in the literature, nor can one be determined by this study. However, compared to examinations in the course of therapy, differences were clearly observable (of 18 patients which received additional radiological imaging, 12 patients received at least one DSA and 12 received at least one CTA in the course, enabling progressional assessment of the vessels width) and in 4 patients abdominal CT examinations with arterial phased vessel contrast were acquired before the clinical suspicion of NOMI arose. Therefore, qualitative criteria as used in this study prove more important.

**Fig 1:**
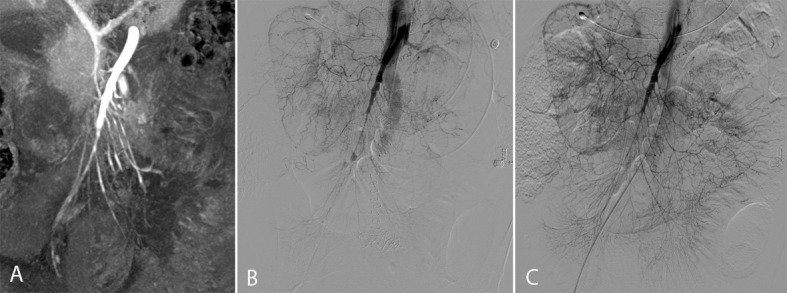
Non-occlusive mesenteric ischemia before and after endovascular vasospasm therapy. Reduced arterial diameter and caliber irregularities in the middle segment and branches of the SMA in CT angiography due to vasospasms from non-occlusive mesenteric ischemia (A). DSA (B) confirms the CT finding. Note the retrograde flow of the contrast agent into the aorta caused by increased vascular resistance in the SMA. Caliber irregularities are reduced after endovascular vasospasm therapy with i.a. prostavasin (C).

Visual assessment of vessel contrast was found to be one of the significant imaging criteria for NOMI in MSCT. Qualitative determination of vessel diameters also appears to be an appropriate method for assessing NOMI, producing similar results compared to the quantitative measurements in the middle and distal segments. Due to the small peripheral vessel diameters and additional vasoconstriction, as a pathophysiological correlate for NOMI, qualitative measurements reveal a higher variance, mostly with respect to the distal vessel segments. Graduation of the severity of NOMI with a progressive decrease in arterial and venous blood vessel enhancement cannot be derived from the results of this study, but Fig 1(A), 1(B) and 1(C) illustrates the reduced arterial vessel width and contrast enhancement as an indicator for progressive deterioration of NOMI.

Nakamura et al. demonstrated that the diameter of the mesenteric vein, relative to the mesenteric artery, can be used as a surrogate parameter for mesenteric blood perfusion by indicating restrictions in arterial inflow, and thus could be used as an appropriate parameter for assessing non-occlusive mesenteric ischemia [16]. However, this was not confirmed by the results of this study. Compared to the other parameters that were examined, the diameter of the mesenteric veins was not a sufficient indicator for diagnosing non-occlusive mesenteric ischemia.

Other secondary signs—examined separately—were deemed insufficient for diagnosing NOMI in MSCT. Other reports suggest that they could offer greater confidence in the diagnosis [24, 27, 28] of NOMI, but in the authors’ opinion cannot be regarded as a single specific indicator. A definite, but small (R1 = 0.31; R2 = 0.29) and not significant (R1 p = 0.10; R2 p = 0.12) correlation was found for reduced contrast enhancement of the bowel wall, as the best of the secondary signs.

There was only a low incidence of imaging findings in this study that indicated bowel wall damage such as intestinal pneumatosis or portal gas. In case of clinically suspected bowel wall damage, CT imaging is performed to detect complications (e.g., free peritoneal gas or fluid indicating bowel wall perforation) and surgery is performed immediately on identification of necrotic bowel segments. These patients therefore did not undergo DSA and thus were not included in the study. Nevertheless, the therapeutic decision in these cases is likewise based on CT imaging, with CT also permitting detection of severe abdominal complications. Additionaly, as postulated by Mazzei et al., CT could reveal the different appearances of NOMI upon imaging depending on the time at which the CT examination is performed and thus, on presence or absence of reperfusion. [27]

In summary, the results of this study demonstrated that MSCT is a helpful method in the diagnostic investigation of suspected NOMI.

Several papers already suggested that CT, with its advanced technical capabilities, will become increasingly important, but most evaluated only small numbers of patients (< 36 patients) [16, 18, 22, 29] or discussed angiography by focusing on interventional treatment for NOMI [16, 29–31]. Other studies reported on the impact of MSCT in the diagnosis of NOMI only in a limited number of patients [32, 33].

Our study evaluates the diameters of the mesenteric artery and the mesenteric vein and the subjective rating of vessel diameter along with secondary CT signs of intestinal damage. The diagnostic decision is based on these parameters. Measuring vessel diameters with the aid of appropriate software tools may improve such assessments in future software generations. The subjective rating of vessel diameter by experienced radiologists appears to be the best method at present.

As the results of this study demonstrate, CT is reliable in almost all cases for non-invasive diagnosis of NOMI and afford advantages over DSA due to detection of potential abdominal complications such as bowel wall perforation with intestinal pneumatosis, portomesenteric gas or even intraperitoneal free air [12, 18, 33–36]. However, DSA is still necessary as an interventional treatment for NOMI after CT diagnosis if medical conditions necessitate immediate therapeutic intervention. Furthermore, the elaborate set of DSA equipment and specialized human resources mean that its distribution is limited.

Thus, with respect to a clinical suspicion, other potential medical causes, the severity of NOMI and other severe diseases requiring immediate therapeutic interventions, the clinical examination of patients suspected to have NOMI should include CT imaging at centers that have an angiography unit, and especially at centers where DSA is not available.

### Limitations

In addition to imaging, the diagnostic investigation of NOMI is based on clinical examination and serum parameters such as lactate and intestinal fatty acid binding protein (I-FABP). The aim of this study was to evaluate the performance of MSCT in patients suspected to have NOMI, and to determine the significant imaging criteria for NOMI in MSCT. For this purpose, MSCT was compared to DSA, which in each case was performed as soon as possible after CT to avoid any changes in hemodynamics that could influence the evaluation. Nevertheless, this study is limited to the extent that DSA is still the “gold standard”—by definition—for angiography of the mesenteric arteries. As elaborated above, the professional experience is essential in ensuring reliable image analysis, especially performing DSA examinations or evaluating cardiovascular CT- and MRI-examinations possibly play an important role in the certainty of reporting, although not specifically evaluated in our study.

Furthermore, severe cases with structural bowel wall damage received primary surgical treatment by necrosectomy and were scheduled for DSA after surgery if deemed necessary by surgical examination during laparotomy. These cases were not included in the study. Consequently, most cases included in our study were less severe but more complex. However, as mentioned above, the detection of severe complications in patients with NOMI is one of the great advantages of MSCT, besides non-invasiveness, compared to DSA.

## Conclusion

MSCT, when evaluated by an experienced reader, is an appropriate method for non-invasive diagnosis of patients with NOMI, permitting adequate and immediate therapeutic stratification.

## Supporting information

S1 Table **S1a (Sheet 1) Data Collection: Imaging.** Sheet 1 of Table 1 contains information on the available examinations (CT and DSA), a therapy, if available, and the temporal context of the therapy. **S1b (Sheet 2) Evaluation CT/DSA.** Sheet 2 of Table 1 contains information on the vessel diameters determined by means of DSA and CT, as well as the indirect CT-signs.
(XLSX)
